# Relative contributions of large-scale and wedgelet currents in the substorm current wedge

**DOI:** 10.1186/s40623-020-01234-x

**Published:** 2020-07-20

**Authors:** Y. Nishimura, L. R. Lyons, C. Gabrielse, J. M. Weygand, E. F. Donovan, V. Angelopoulos

**Affiliations:** 1 Department of Electrical and Computer Engineering and Center for Space Physics, Boston University, Boston, MA USA; 2 Department of Atmospheric and Oceanic Sciences, University of California, Los Angeles, CA USA; 3 The Aerospace Corporation, Los Angeles, CA USA; 4 Department of Earth, Planetary and Space Sciences, University of California, Los Angeles, CA USA; 5 Department of Physics and Astronomy, University of Calgary, Calgary, AB Canada

**Keywords:** Substorm current wedge (SCW), Field-aligned current (FAC), Westward traveling surge (WTS), Plasma sheet, Bursty bulk flow (BBF)

## Abstract

We examined how much large-scale and localized upward and downward currents contribute to the substorm current wedge (SCW), and how they evolve over time, using the THEMIS all-sky imagers (ASIs) and ground magnetometers. One type of events is dominated by a single large-scale wedge, with upward currents over the surge and broad downward currents poleward-eastward of the surge. The other type of events is a composite of large-scale wedge and wedgelets associated with streamers, with each wedgelet having comparable intensity to the large-scale wedge currents. Among 17 auroral substorms with wide ASI coverage, the composite current type is more frequent than the single large-scale wedge type. The dawn–dusk size of each wedgelet is ~ 600 km in the ionosphere (~ 3.2 *R*_E_ in the magnetotail, comparable to the flow channel size). We suggest that substorms have more than one type of SCW, and the composite current type is more frequent.
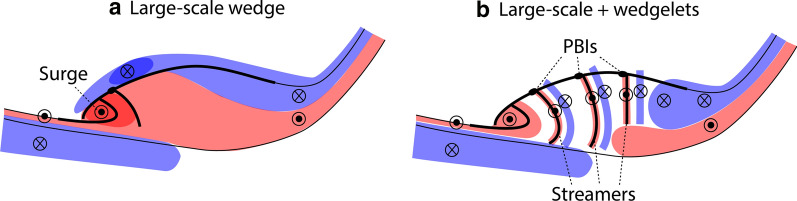

## Introduction

The substorm current wedge (SCW) characterizes the major current system during the substorm expansion phase (Rostoker 1968, 1969; Meng and Akasofu 1969; McPherron et al. 1973). The westward electrojet creates a negative deflection of the *H*-component of the ground magnetic field at auroral latitudes (negative bay). It is driven by magnetospheric forces through a pair of upward and downward field-aligned currents (FACs) that are separated azimuthally and create positive *H*-deflections (positive bay) at mid and low latitudes. The SCW begins at a small region soon after substorm auroral onset and then expands poleward and azimuthally, forming the auroral bulge. The expansion is often not smooth but progresses in a stepwise manner (Kisabeth and Rostoker 1974).

Traditionally, the SCW has been considered to be a large-scale, wedge-shaped current system (McPherron et al. 1973; Gelpi et al. 1987; Connors et al. 2014; Chu et al. 2014). The SCW model has successfully explained various substorm-related features such as averaged ionospheric current and convection patterns (Kamide et al. 1996) and the polarization of Pi2 pulsations (Lester et al. 1989). Variants of the SCW model include multistep intensifications with westward and poleward leaps (Wiens and Rostoker 1975), and a secondary loop for region-2 (R2) FACs (Ritter and Lühr 2008).

On the other hand, a number of studies have reported possible signatures of multiple localized currents (wedgelets) that are separated azimuthally by hundreds of km (Sergeev 1974; Pytte et al. 1976; Rostoker 1991; Kauristie et al. 2000; Lyons et al. 2012; Forsyth et al. 2014; Liu et al. 2015; Palin et al. 2015). Wedgelets are embedded in the SCW and form multiple wedge-type currents located across the longitude range of the substorm bulge. Recent MHD simulations have reproduced wedgelets associated with formation of multiple fast flow channels (bursty bulk flows (BBFs)) in the magnetotail (Birn et al. 2019; Merkin et al. 2019). Each wedge is more localized and has a shorter duration than the SCW (Pytte et al. 1976).

The strongest upward FACs exist over the auroral surge (folds forming at the western edge of the bulge) (Opgenoorth et al. 1983; Fujii et al. 1994; Partamies et al. 2003), and the westward expansion of the upward FACs corresponds to the westward traveling surge (WTS). However, it is often uncertain where the downward FACs are located and how large the separation between upward and downward FACs is. In the conventional SCW model, derived principally from mid-latitude and geosynchronous observations, the downward current is located at post-midnight. In contrast, low-altitude satellite observations suggest that a large fraction of the surge current closes locally (Marklund et al. 2012), and that downward FACs are broadly distributed just poleward of the upward FACs and auroral oval (in the so-called R0 current system) (Fujii et al. 1994; Gjerloev and Hoffman 2002). Peak location of the R0 downward currents is shifted by only ~ 1 h MLT downward of the upward FACs, and the imbalance of upward and downward FACs at each MLT is related to the westward electrojet. Alternating polarities of FACs are found in the bulge, suggesting localized FAC structures (Gjerloev and Hoffman 2002; Forsyth et al. 2014; Kepko et al. 2015). However, it is unclear whether those currents have coherent structures (showing localized wedges separated azimuthally) or are randomly distributed (as illustrated in Kepko et al. 2015), and what their spatial extent is. It is also not quantitatively understood how much currents wedgelets contribute to the SCW. Other important questions are whether all substorms have the same type of current structures, and if not, which type of currents (dominated by a single large-scale wedge, dominated by wedgelets, or a combination of both) occurs more frequently.

To address those questions, we examine the 2-D structure and dawn–dusk size of substorm upward and downward currents and related aurora, using the spherical elementary currents systems (SECS) method (ground magnetometers) (Weygand et al. 2011) (with an error analysis conducted by Weygand and Wing 2016) and Time History of Events and Macroscale Interactions during Substorms (THEMIS) ASIs (Mende et al. 2008). The ASIs can resolve ~ 10 km auroral structures in 3-second cadence, and the SECS method provides vertical currents from divergence of the curl-free system from ground magnetometers as a proxy of FACs with a ~ 200 km and 10 s resolution. As in the references in this section, ground magnetometers have been widely used for deducing the 3-D SCW and wedgelet current system of substorms. However, examination of current structures covering a wide longitude range has been rare.

We identified auroral substorm events where the THEMIS ASIs detected substorm auroral onset (initial brightening followed by poleward expansion (Akasofu 1964)). To remove pseudo-breakups and weak substorms, we required that poleward expansion continued for several minutes or longer, and that the SuperMAG *AL* (*SML*) index reached below −300 nT. We also required that substorm onset occurs in Central Canada, and that the ASIs and magnetometers fully cover the substorm bulge under dark, clear sky conditions. In the existing dataset since 2008, we found 17 substorms that satisfy these conditions (2008-3-5 6 UT, 2008-3-11 5 UT, 2008-3-28 5 UT, 2008-10-2 5 UT, 2008-12-31 5 UT, 2010-2-16 7 UT, 2011-3-3 7 UT, 2011-4-2 6 UT, 2011-4-9 5 UT, 2011-10-2 5 UT, 2012-2-13 6 UT, 2013-10-30 7 UT, 2014-2-24 8 UT, 2014-2-28 7 UT, 2014-3-3 6 UT, 1015-10-16 7 UT, and 2017-3-1 5 UT). The requirement of wide ASI coverage limits the number of events but allows us to be certain that the substorms are covered by the ASIs. The requirement on the multiple imager availability only gave a limited number of events in the existing dataset. However, these selection criteria are not related to structures of currents or aurora within the substorm region, and thus do not create a bias in selection of the types of events. We first present two representative events, and then we describe the dawn–dusk size of all events.

## Results

### Time sequence

**Fig. 1:**
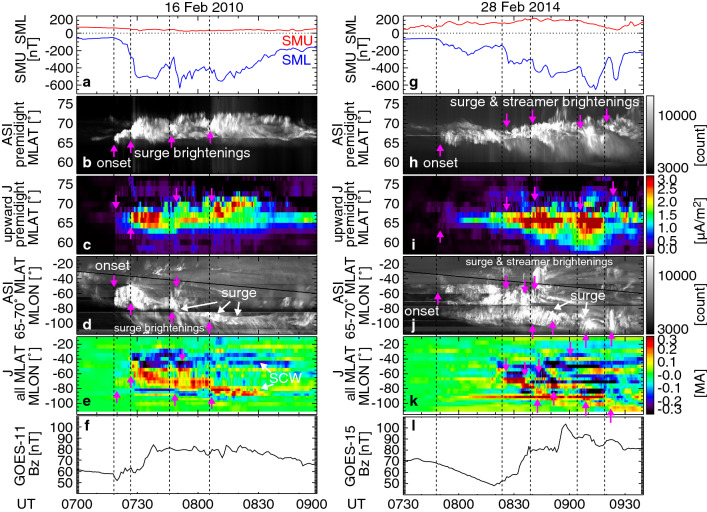
*SMU*/*SML* indices, ASI north–south keogram at pre-midnight, maximum upward current north–south keogram at each latitude at pre-midnight, ASI east–west keogram at 65°–70° MLAT, current east–west keogram integrated over latitude, and GOES magnetic field. **a**–**f** and **g**–**l** are for the 16 February 2010 and 28 February 2014 substorms. GOES-11 and 15 are about −70° MLON and reach midnight at 9 UT. The dashed lines and pink arrows highlight auroral and current enhancements. The solid line in Panels d and j marks the magnetic midnight meridian

Figure 1 compares two substorm events, where both events had a similar level of minimum *SML* and occurred at similar UT and day of year. *SML* for both events for the preceding > 3 h was above −100 nT, and the substorm auroral onset latitudes were nearly at the same latitude. However, as shown below, these events had different types of current behavior. Figure 1b and h shows auroral north–south keograms depicting, the maximum intensity at each latitude in each time over 21–24 MLT (see “2-D distribution” section for 2-D spatial distribution). This range of MLT was used to construct the keogram in order to identify all major auroral brightenings occurring at various MLTs in one panel. The aurora was quiet initially, and then substorm auroral onset occurred at 7:18 (Fig. 1b) and 7:48 UT (Fig. 1h), followed by poleward expansion and *SML* enhancement. The aurora and *SML* intensified multiple times during the substorm development, as indicated by the dashed lines and vertical arrows. Such multiple intensifications are a typical sequence during substorms (McPherron 1979). The aurora during the expansion phase shows multiple intensifications of roughly north–south oriented auroral arcs that moved equatorward (auroral streamers); these are typical auroral features during substorms (Rostoker et al. 1987; Sergeev et al. 2004). Figure 1c and i shows maximum upward current keograms versus latitude at 21–24 MLT from the SECS method. The upward currents intensified multiple times nearly at the same latitudes and times as aurora. This indicates that the SECS method properly found upward currents associated with the major auroral intensifications. Although SECS cannot resolve currents associated with small-scale (< 200 km) and short-lasting (< 10 s) auroral intensifications, currents associated with major *SML* enhancements can be detected and are the focus of this study. The second event developed more gradually over time, and the oval extended further equatorward.

The east–west patterns show striking differences. Similar to Fig. 1b and h, the east–west keogram were created by calculating the maximum intensity over 65°–70° deg MLAT (where much of intense auroral activity occurred) at each longitude and time. In the first event, the aurora propagated westward (WTS, see also “2-D distribution” section) and the multiple intensifications occurred in the vicinity of the WTS (Fig. 1d). The aurora at more eastern and western longitudes was much dimmer, except for a brief intensification at 07:50–07:58 UT. The currents integrated over all available latitudes (55°–80° MLAT) at each longitude are shown in Fig. 1e. The latitudinal integration removes currents that are balanced in the north–south direction (not contributing to the westward electrojet or mid-latitude positive bays), so that we can extract currents that are connected to the westward electrojet and hence the *SML* intensifications. The SCW can be clearly identified as a pair of upward current (red) to the west, and downward current (blue) to the east, which extends over the substorm bulge longitudes. The upward current propagated westward in association with the WTS and intensified together with the WTS brightenings (moving from −60° to −90° MLON). The dawn–dusk distance between the upward and downward currents increased over time from ~ 1.3 MLT width at 7:30 UT to ~ 2.7 MLT width at 8:10 UT, indicating the growing dawn–dusk size of the SCW (Kisabeth and Rostoker 1974; Chu et al. 2014).

In contrast, the dawn–dusk pattern of aurora and currents in the second event was much more structured (Fig. 1j, k). Although the WTS can be seen at 8:30–8:50 UT (moving from −80° to −110° MLON), the aurora at other longitudes were as intense as the WTS and brightened multiple times at various longitudes. Similarly, the currents showed a composite of large-scale SCW and wedgelet structures. The wedgelet currents were as intense as the large-scale currents and dynamically evolved over time. Many of the intensifications seems to initiate at (or near) the poleward boundary of the auroral oval, and thus initiate as what have become known as poleward boundary intensifications (PBIs).

The last panels show the geosynchronous *B*_z_ magnetic field measured by GOES-11 (Fig. 1f) and GOES-15 (Fig. 1l), both of which were at midnight at 9 UT. Figure 1f shows essentially only one major dipolarization at 07:30–07:40 UT (other small variations also exist), while Fig. 1l shows two-step (08:30–08:40 and 08:50–09:00 UT) or potentially more dipolarizations. This behavior is consistent with the aurora and currents, where the first event had a single active auroral region (surge) that moved duskward, and the second event had multiple intensifications near midnight.

### Mid-latitude ground magnetic field

**Fig. 2:**
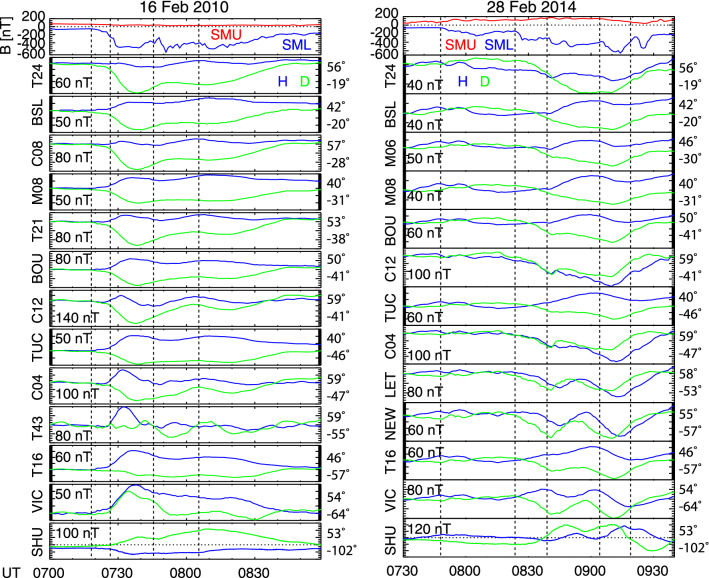
The top panels are the *SMU*/*SML* indices for the 16 February 2010 and 28 February 2014 events. The other panels show the *H* (blue) and *D* (green) components of mid-latitude magnetometer data, sorted by magnetic longitude. The magnetic latitude and longitude are indicated to the right of each panel. The *y*-scale for each panel is indicated in units of nT

The difference in the SCW structure and evolution can also be inferred independently from mid-latitude magnetometer data. Figure 2 plots the *H*- and *D*- component magnetometer data from selected stations located in North America (−102° to −19° MLON) at 40°–60° MLAT. In the first event, almost all magnetometers detected positive bays associated with the first three *SML* enhancements, although timings of the *H*-component peaks depend on longitude, because the distance between the currents and magnetometers changes over time. The westernmost station (Shumagin, SHU) measured negative deflections in the first two pulses (outside SCW longitudes), and then positive deflections (in SCW longitudes). The eastern stations measured negative deflections as the surge traveled westward. The *H*-component changed the signs between SHU and VIC (Victoria), and was nearly zero at C08 (Osakis) and T24 (Shawano). The longitudinal range of the positive *H* is comparable to the longitudinal extent of the currents found in the SECS method in Fig. 1e (−90° to −30° MLON). The positive bays for the fourth SML enhancement were detected only by a few westernmost stations because the auroral activity shifted westward. The *D*-component deflection was the smallest at T16 (Carson City, −57° MLON), indicating the proximity to the central longitude of the SCW. This is consistent with the SECS method in Fig. 1e, where the center of the SCW is inferred to be ~ −60° MLON. All these magnetometer responses are consistent with the classical large-scale SCW model (McPherron et al. 1973).

The second event does show positive bays over a wide longitude range suggesting a large-scale SCW, indicating the existence of the SCW. The *H*-component was small at SHU (−102° MLON) and T24 (−19° MLON). These longitudes are roughly consistent with the longitudes of the westernmost upward and easternmost downward currents in the SECS method (Fig. 1k), indicating the SCW size. However, the positive bays across longitude are not as coherent as in the first event. Only a few stations at nearby longitudes showed a positive bay for each *SML* enhancement, but the magnetometers at other longitudes measured markedly different variations. For the 8:40 UT electrojet enhancement, the *D*-component deflection was small at T16, TUC (Tucson, −46° MLON), and M08 (San Antonio, −31° MLON). Also two stations that are at the same longitude but at different latitudes [BOU (Boulder) and C12 (Weyburn)] showed different magnetic field records. According to Biot–Savart’s law, magnetic field due to localized currents decays more rapidly with distance from the currents. This suggests that the mid-latitude magnetic fields cannot be solely explained by a large-scale wedge current system but are influenced by wedgelets, consistent with our findings from aurora and currents in “Time sequence” section. The positive deflections peaking around 09:00 UT were detected by most stations, but as can be seen in Fig. 1k, this was due to wedgelets rather than a single large-scale wedge.

### 2-D distribution

**Fig. 3:**
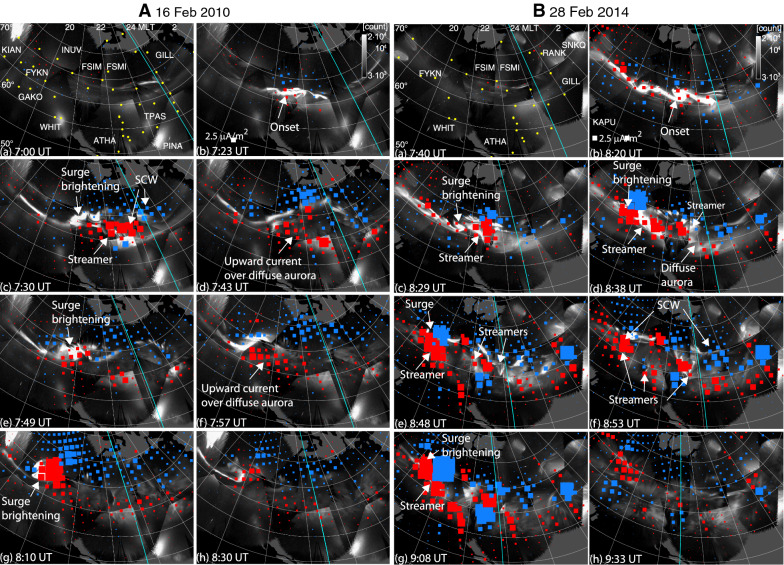
Selected images of upward (red) and downward (blue) currents overlaid onto ASI data (grayscale) for the (**A**) 16 February 2010 and (**B**) 28 February 2014 event. The light-blue line marks magnetic midnight. The latitude and longitude contours are given every 5° and 15°. Magnetometer locations are marked by yellow dots in Panel a. The whole sequence is given in Additional files 1 and 2

To discuss the 2-D structure of the aurora and currents for these two events, we plotted the currents over the THEMIS ASI mosaics as shown in Fig. 3. The entire temporal sequence can be seen in Additional files 1 and 2. The locations of the magnetometers used for the SECS method are shown by the yellow dots in Panel a. The SECS method cannot find currents narrower than magnetometer separations (< ~200 km), but the regions with the strong currents are overall covered by the magnetometers, and auroral brightenings related to the major *SML* enhancements shown below are comparable to or larger than a few 100 km. A coverage gap, however, exists between Athabasca (ATHA) and Whitehorse (WHIT), and the current in this region is less reliable.

In the first event, Fig. 3Ab, c, e and g correspond to the major auroral brightenings in Fig. 1. The first brightening is the substorm auroral onset along the pre-existing arc. The subsequent three brightenings are the surge and the streamers to the east of the surge. Upward currents peaked in association with those brightenings. Corresponding downward current enhancements were located about 500–1000 km northeast of the upward currents. This pair of time-varying upward and downward currents constitutes a wedge-type current system at the spatial size of the substorm bulge, and hence a SCW. Part of the downward currents extended poleward of the auroral bulge. The current distribution is consistent with the findings by Tighe and Rostoker (1981), Fujii et al. (1994) and Gjerloev and Hoffman (2012), and the downward currents extending poleward of the auroral oval is similar to the R0 currents. When the aurora weakened after each brightening (Fig. 3Ad, f and h), the peak upward currents decreased and shifted away from the surge toward diffuse aurora southeastward of the surge. Morphologically, those currents appear to connect to the upward R2 currents in the post-midnight sector. The existence of diffuse aurora suggests that these enhanced upward currents could represent enhanced R2 currents due to a buildup of the near-Earth plasma sheet plasma pressure. The downward currents are mainly poleward of the upward currents and appear to be connected to the post-midnight downward R1 currents. However, such currents were seen when *SML* was decaying, and the upward and downward currents were separated predominantly in the north–south direction. As opposed to the primarily east–west separation for the currents over the surge, the north–south separation of the currents only has minor contributions to positive bays at mid-latitudes.

Thus the upward currents of the SCWs in the February 16 event are mainly located over the surge and streamers, and expand northwestward in a stepwise manner. This evolution is consistent with the aforementioned steplike expansion of auroral electrojets (Wiens and Rostoker 1975). Because the surge and streamers are indicative of plasma sheet flow bursts (Liu and Rostoker 1993; Sergeev et al. 2004; Mishin 2016), these observations suggest that the SCW is directly related to the plasma sheet flow bursts (Keiling 2009). In contrast, the currents over the diffuse aurora after each auroral brightening are not a major part of the SCW, and thus the plasma and magnetic flux piling up in the near-Earth plasma sheet following flow bursts do not likely directly contribute to the SCW currents. The enhanced upward and downward currents are not exactly separated in east–west, but are tilted to northeast–southwest. Moreover, the north–south size of the currents is comparable to the east–west separation of the currents. In this current configuration, positive bays at low-mid latitudes are not confined within the SCW longitudes but spread over wider longitudes at lower latitudes than at auroral and mid-latitudes. This is consistent with an inference from positive bay observations by Lester et al. (1989).

In the second event, five major auroral brightenings are shown in Fig. 3Bb–e and g. Similar to the first event, peak upward currents were found in association with bright discrete aurora. Although part of the enhanced upward currents was seen over the WTS, comparably intense upward currents can be seen at other longitudes over bright discrete aurora. Most of those corresponded to auroral streamers, and the others occurred over diffuse aurora. The SCW can be identified as an overall upward currents at pre-midnight and downward currents at post-midnight, but the currents in the bulge had wedgelets that were equally intense. The currents are highly structured but more organized than illustrated in Fig. 9 of Kepko et al. 2015. Downward currents were located northeastward of the upward currents in each wedgelet, with a narrower separation (~ 200–300 km) than in the first event (~ 500–1000 km). Such a localized current configuration has been detected for auroral streamers using ground magnetometers (Amm et al. 1999).

The wedgelets were comparable in intensity to the large-scale wedge, and thus the wedgelets are a critically important component of the substorm current in this event. The northwestward expansion of the SCW in this event also occurred in a stepwise manner but the surge brightenings occurred as new, localized currents associated with streamers (azimuthally displaced from the prior activity) rather than intensifications and expansions of a pre-existing, large-scale SCW with a single surge. This evolution is similar to the results by Pytte et al. (1976), where a separate SCW occurs to the east of the preceding SCW and propagate westward.

### Multi-event analysis of the dawn–dusk size

We have calculated east–west distribution of currents for all 17 events in the same way as for Fig. 1e and k. The number of events is limited due to the selection criteria and we do not aim at finding firm statistical properties. We limit ourselves to characterize properties among these events. To characterize the dawn–dusk size of the currents, we conducted a fast Fourier transformation (FFT) analysis for the currents near the end of the substorm expansion phase (maximum |*SML*|). The choice of the time is made to obtain the maximum dawn–dusk size after the bulge stopped expanding. Other choice of times would underestimate the size and make it difficult to compare different events.

**Fig. 4:**
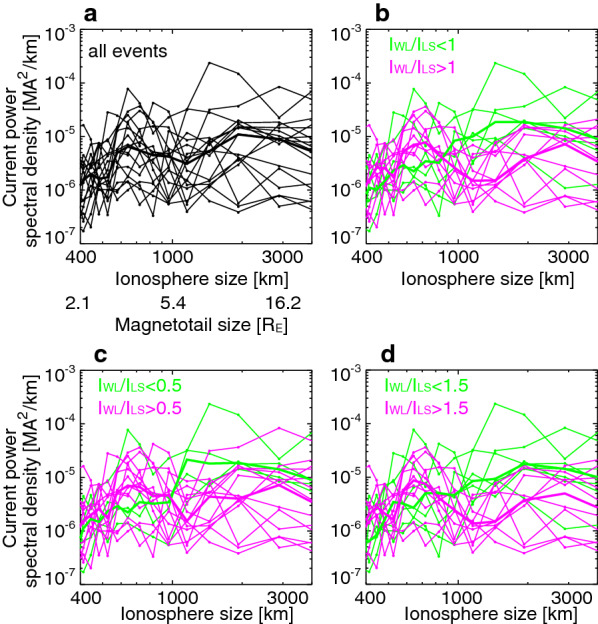
**a** Azimuthal spectra of the upward/downward currents integrated over latitude. The dots and thin lines correspond to individual events, and the thick line shows the median. **a** All events. **b** Same as Panel a but color-coded with $I_{\text{WL}} /I_{\text{LS}} > 1$ (pink) and $I_{\text{WL}} /I_{\text{LS}} < 1$ (green). **c** Same as Panel a but color-coded with $I_{\text{WL}} /I_{\text{LS}} > 0.5$ (pink) and $I_{\text{WL}} /I_{\text{LS}} < 0.5$ (green). **d** Same as Panel a but color-coded with $I_{\text{WL}} /I_{\text{LS}} > 1.5$ (pink) and $I_{\text{WL}} /I_{\text{LS}} < 1.5$ (green). $I_{\text{WL}}$ and $I_{\text{LS}}$ are peak power spectral density below 1000 km (indicator of the wedgelet component) and above 1000 km (indicator of the large-scale component) wavelengths

**Fig. 5:**
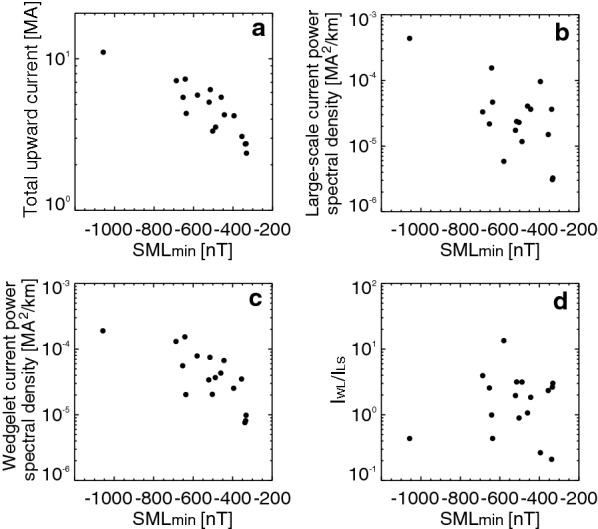
**a** Maximum total upward current, **b**$I_{\text{LS}}$, **c**$I_{\text{WL}}$, and **d**$I_{\text{WL}} /I_{\text{LS}}$ as a function of the minimum *SML* for individual events

The current power spectra for all events are shown in Fig. 4a as a function of the dawn–dusk wavelength in the ionosphere. The thick line shows the median. Within the measurable wavelength scale (> 400 km), the spectral peak at ~ 2000–3000 km corresponds to the whole dawn–dusk extent of the SCW (large-scale wedge). To estimate how many events show a substantial spectral peak corresponding to the wedgelet component, we color-coded the events according to the ratio between peak intensities below 1000 km ($I_{\text{WL}}$) and above 1000 km ($I_{\text{LS}}$) wavelength. The 1000 km wavelength corresponds to a 1.4 MLT size at 65° MLAT. Figure 4b uses $I_{\text{WL}} /I_{\text{LS}} = 1$ as a threshold. Eleven out of the 17 events (65%) have a peak above $I_{\text{WL}} /I_{\text{LS}} = 1$. For the rest of the events (6 events, 35%), as the median line shows, the spectra below 1000 km wavelength show an overall decrease toward smaller wavelengths. The percentages depend on the threshold and would also be more accurate if we were to examine a larger number of events. But among the events we have, different thresholds also show that events with the significant wedgelet component occurs more often. For example, $I_{\text{LS}}$/$I_{\text{WL}} = 0.5$ (Fig. 4c) gives 13 and 4 events above and below the threshold, and $I_{\text{LS}}$/$I_{\text{WL}} = 1.5$ (Fig. 4d) gives 10 and 7 events above and below the threshold. The $I_{\text{WL}} /I_{\text{LS}}$ values for all events are shown in Fig. 5d.

We roughly estimated the magnetotail size of these activations using the T01 magnetic field model (Tsyganenko 2002) by taking the solar wind and SYM-H parameters of Fig. 1a–f event as a representative event. The model does not consider substorms and mapping variations among events, but here we intend to provide approximate information without pursuing high accuracy. The field-line at 66° MLAT and 23.2° MLT in the ionosphere near the center of the SCW reaches 14.2 *R*_E_ at 23.5 MLT. The azimuthal mapping factor (the ratio between the magnetosphere and ionosphere azimuthal separations of two adjacent field lines) is 34 and is used to give the magnetotail size in the bottom of Fig. 4a.

The localized currents have a typical magnetotail dawn–dusk size of ~ 3.2 *R*_E_, and the large-scale currents have a size of > ~ 10 *R*_E_. The ~ 3.2 *R*_E_ size is comparable to the typical size of BBFs (Angelopoulos et al. 1997; Nakamura et al. 2004), and thus is consistent with the idea that BBFs are linked to localized and transient currents in the ionosphere. This implies that the localized and transient currents likely associated with BBFs are dominant contributors to the substorm currents for many of the substorms.

Figure 5 shows current parameters as a function of the minimum *SML* of individual events. Figure 5a plots the maximum total upward current, and confirms that the currents obtained from the SECS method are proportional to the substorm strength and gives typical values (Anderson et al. 2018). Figure 5b, c shows $I_{\text{LS}}$ and $I_{\text{WL}}$ for each event that are obtained from Fig. 4a. $I_{\text{LS}}$ and $I_{\text{WL}}$ are also proportional to the substorm strength. In contrast, $I_{\text{LS}} /I_{\text{WL}}$ does not have a clear dependence on the substorm strength (Fig. 5d). This indicates that, among the events we have, the occurrence of substorms with strong wedgelets is not controlled by the amount of energy that is released from the magnetotail. The wide range of $I_{\text{LS}} /I_{\text{WL}}$ suggests that the contribution of wedgelets in substorm currents is highly variable.

Currently it is unclear what controls $I_{\text{LS}} /I_{\text{WL}}$. We have also checked solar wind parameters and the SYM-H index, but we did not find a clear relation between those parameters and $I_{\text{LS}} /I_{\text{WL}}$ (not shown). For example, both events in Fig. 1 occurred under IMF *B*_z_ ~ −5 nT. Those two events occurred during the storm recovery phase (SYM-H ~ −40 and −70 nT), but not all events occurred during storms.

## Conclusion

**Fig. 6:**
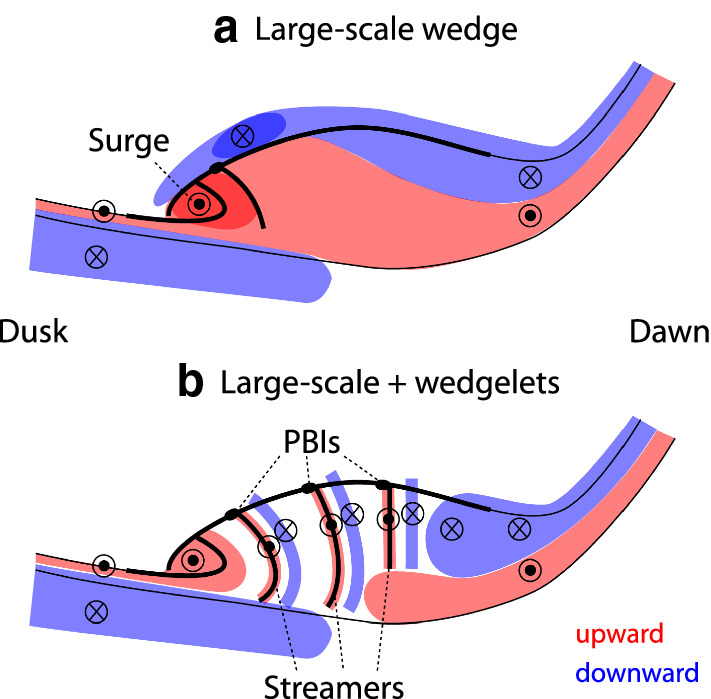
Illustration of upward (red) and downward (blue) currents for events dominated by **a** the large-scale wedge and **b** a composite of the large-scale and wedgelet currents. The thin black lines mark the poleward and equatorward auroral boundaries. The thick lines mark bright auroral arcs

We have determined the dawn–dusk structure of latitudinally integrated currents using the THEMIS ASIs and ground magnetometers, and discussed the detailed evolution of the upward and downward currents. We identified two major types of current structures. Currents of the first type of substorm (Figs. 1a–f, 6a) are dominated by a single large-scale wedge. The WTS and adjacent streamers are the most dominant auroral structures. Most intense upward currents propagate westward in association with the WTS, and the downward currents are located in a broad region northeast of the upward currents. The aurora and currents intensify multiple times in the WTS region, and the dawn–dusk separation between the upward and downward currents increases over time. Mid-latitude positive bays within the SCW meridians evolve coherently across a wide longitude range, supporting the presence of a large-scale SCW.

Currents in the second type of substorms (Figs. 1g–l, 6b) are characterized by a composite of large-scale and wedgelet upward and downward currents. The WTS exists in association with upward currents, which could be the large-scale component in common with the single large-scale wedge-type events, although the WTS in the second type of substorms is not as stable or dominant. However, aurora and upward currents at other longitudes are comparably intense, and enhanced upward currents tend to be found over bright auroral streamers. Each activation only lasts for ~ 10 min, and multiple and transient intensifications occur at various longitudes in the nightside auroral oval. Similarly, the mid-latitude positive bays do not show a high correlation across longitudes, also indicating the existence of wedgelets.

The composite current events are more frequent than the events with a single large-scale wedge. The dawn–dusk size of the large-scale wedge is a few 1000 km in the ionosphere, which corresponds to ~ 10 *R*_E_ in the magnetotail. The dawn–dusk size of each wedgelet is ~ 600 km in the ionosphere, ~ 3.2 *R*_E_ in the magnetotail. This size is comparable to the typical size of BBFs. These results suggest that substorms exhibit more than one type of aurora and current distributions, but the currents are frequently contributed by wedgelets. The currents of the wedgelets are as intense as the large-scale wedge currents. Localized magnetotail reconnection and BBFs likely play a major role in creating the wedgelets. In the rest of the events, BBFs seem to be weaker or more confined to the WTS region, and the aurora and currents follow the traditional large-scale SCW pattern. However, such events are less common than those with multiple, localized currents.

We note that, although the SECS method provides 2-D distributions of currents that are consistent with the auroral structures, the derivation of the currents is based on the assumptions that the Hall-to-Pedersen conductance ratio is constant and that the conductance gradient perpendicular to the electric field is negligible (Amm et al. 2002). These assumptions may be violated in areas with sharp luminosity gradients. It is difficult to obtain conductance distributions with coverage and resolution that are comparable to SECS, and thus it is not possible to evaluate how much the conductance gradients may affect the current estimates. These conclusions are based on a limited number of events where multiple imagers were available and detected the entire longitudinal extent of the substorm bulge. More accurate statistics would require investigating more events and using different thresholds.

## Supplementary information

**Additional file 1.** THEMIS ASI snapshots on 16 February 2010. The format is the same as in Fig. 3. The images are given every 15 s.

**Additional file 2.** THEMIS ASI snapshots on 28 February 2014. The format is the same as in Fig. 3. The images are given every 15 s.

## Data Availability

THEMIS ASI, SECS and magnetometer data were obtained through https://themis.ssl.berkeley.edu, vmo.igpp.ucla.edu, and supermag.jhuapl.edu.
